# Phase II trial of pembrolizumab, ipilimumab, and aspirin in melanoma: clinical outcomes and translational predictors of response

**DOI:** 10.1038/s44276-024-00057-7

**Published:** 2024-06-24

**Authors:** Zoe Quandt, Saya Jacob, Muhammad Zaki Hidayatullah Fadlullah, Chaorong Wu, Clinton Wu, Laura Huppert, Lauren S. Levine, Paula Sison, Katy K. Tsai, Melissa Chow, Jee Hye Kang, Jimmy Hwang, James C. Lee, Ariel Oglesby, Jessica Venegas, Ben J. Brintz, Aik Choon Tan, Mark S. Anderson, Michael D. Rosenblum, Arabella Young, Adil I. Daud

**Affiliations:** 1 Department of Medicine, University of California San Francisco, San Francisco, CA 94143 USA; 2 Diabetes Center, University of California San Francisco, San Francisco, CA 94143 USA; 3 Helen Diller Family Comprehensive Cancer Center, University of California San Francisco, San Francisco, CA 94143 USA; 4 Departments of Oncological Sciences and Biomedical Informatics, University of Utah, Salt Lake City, UT 84112 USA; 5 Division of Epidemiology, University of Utah, Salt Lake City, UT 84112 USA; 6 Huntsman Cancer Institute, University of Utah Health Sciences Center, Salt Lake City, UT 84112 USA; 7 Department of Pathology, University of Utah School of Medicine, Salt Lake City, UT 84112 USA; 8 Dermatology, University of California San Francisco, San Francisco, CA 94143 USA

## Abstract

**Objective:**

Many patients with melanoma treated with immune checkpoint inhibitors (ICIs) do not derive response. Preclinical and retrospective studies identified that inhibition of the cyclooxygenase (COX) pathway may improve response to ICI treatment.

**Methods:**

This prospective single site phase II trial accrued patients with advanced/metastatic melanoma. Participants underwent high-dose aspirin daily combined with pembrolizumab and ipilimumab every 3 weeks for 4 cycles followed by high-dose aspirin and pembrolizumab monotherapy. The primary endpoint was objective response rate (ORR). Longitudinal sampling of blood was performed to assess peripheral immune correlates.

**Results:**

Twenty-seven subjects were enrolled with median follow-up of 32 months. An ORR of 62.9% was reached prior to discontinuation due to low likelihood of achieving the pre-specified ORR of 80%. 17 patients (63%) experienced a treatment-related adverse event (TRAEs) grade 3 or higher. A per-protocol analysis showed that patients able to continue aspirin alongside ICI through the induction period experienced significant survival benefit. Ten cytokines and increased regulatory T cells in the periphery correlated with beneficial response.

**Conclusions:**

The addition of high-dose aspirin to combination ICI within this study results in response comparable to ICI alone. Future clinical studies of COX inhibition will need to focus on mitigation of AEs to establish the clinical utility of this combination.

## Introduction

Immune checkpoint inhibitors (ICIs) have greatly improved the treatment of advanced/metastatic melanoma, particularly when given in combination [1–8]. However, between 40 and 60% of advanced/metastatic melanoma patients do not respond to ICIs, potentially due to additional immunosuppressive mechanisms within the tumor microenvironment (TME) [9, 10].

Tumor-derived immune suppression through the cyclooxygenase (COX) pathway, specifically the production of prostaglandin E2 (PGE2) by the enzyme COX2, has been shown to reduce immune recognition of tumor cells in preclinical models [11]. This is in part due to the enrichment of a regulatory network of cancer-promoting gene targets, such as those that enhance angiogenesis and inhibit immune-mediated cytotoxicity, that correlate with increased COX2 expression in the TME, which may limit anti-tumor immunity [12]. In mouse models, COX2 inhibition in combination with programmed death-1 (PD-1) blockade remodeled the TME and reduced tumor growth [11, 13]. These preclinical studies used both aspirin (an irreversible COX1 and COX2 inhibitor) as well as celecoxib (a COX2 specific inhibitor) in combination with a PD-1 inhibitor and showed rapid tumor regression with both agents, though to a lesser extent with celecoxib as opposed to aspirin [11]. A retrospective study of COX inhibitor use in combination with ICI treatment in melanoma reported longer times to cancer progression [14].

Aspirin has been used to treat skeletal and muscular symptoms as well as primary and secondary prevention of atherosclerotic events, highlighting its potential to be repurposed in the cancer setting. Furthermore, in a randomized trial for patients with Lynch syndrome, a condition mediated by a genetic defect in DNA mismatch-repair genes, aspirin use reduced the incidence of colorectal cancer indicating that aspirin may prevent the development of colorectal cancer in high risk populations [15]. Mechanistically, this anti-cancer benefit may occur at least in part due to limiting tumor promoting chronic inflammation.

Based on this preliminary data, we hypothesized that the addition of high-dose aspirin to ICI therapy would increase response rates in patients with advanced/metastatic melanoma. To test this hypothesis, we conducted the first prospective phase II single-arm study of combination high-dose aspirin with standard of care ipilimumab (a CTLA-4 inhibitor) and pembrolizumab (a PD-1 inhibitor) for patients with advanced/metastatic melanoma to determine safety and efficacy of COX inhibition when combined with ICI.

## Methods

### Study design and subjects

#### Subjects

Patients were eligible for this open-label, prospective, single-arm phase II trial if they had a pathologically confirmed unresectable Stage III or Stage IV melanoma measurable by RECIST 1.1 and adequate organ function. Patients were excluded if they had: (i) uveal melanoma, (ii) received prior anti-PD-1 or anti-CTLA-4 therapy in the metastatic setting or (iii) a history of active autoimmune disease requiring immunosuppressive treatments. The study protocol was reviewed and approved by the institutional review board (**UCSF IRB#CC17854; NCT#03396952**) and all patients provided informed consent.

#### Procedures

Patients received pembrolizumab 200 mg and ipilimumab 1 mg/kg intravenous (IV) on the first day of each three-week cycle for the first four cycles followed by pembrolizumab 200 mg monotherapy IV every three weeks for the duration of the study. Aspirin was taken orally at a dose of 975 mg twice daily for the duration of the study. This dose of aspirin was selected based on preclinical mouse studies with an equivalent dose translated to human patients [11]. For remediation of potential side effects from this high dose of aspirin, patients discussed use of proton-pump inhibitors (PPIs) at the consenting visit. All patients elected to initiate PPIs while on the trial. Patients remained on study until they had progression by imaging or clinical evaluation, experienced unacceptable toxicity or reached a duration of 2 years, whichever came first. Formal clinical assessments of response were completed per RECIST version 1.1 criteria on imaging at baseline and every 12 weeks thereafter (every 4 cycles).

### Data collection

The primary endpoint was the overall response rate (ORR), defined as the proportion of subjects who achieved complete or partial response as their best overall response by RECIST version 1.1. Patients were also marked as having a complete response despite evidence of residual disease on imaging if biopsy of that site showed no evidence of disease. Secondary endpoints included toxicity, as determined by CTCAE version 5, progression-free survival (PFS), defined as time from enrollment until confirmed disease progression by RECIST 1.1, clinical progression or death from any cause, and overall survival (OS), defined as time from enrollment until death from any cause.

#### Correlative studies

Longitudinal sampling of serum and peripheral blood mononuclear cells (PBMCs) from a proportion of patients that consented to research blood collections were used to study cytokine/chemokine differences and changes to immune cell composition.

Simultaneous multiplexed quantification of 71 human cytokines, chemokines, and growth factors was performed using undiluted serum samples run in technical duplicates on the Luminex™ 200 system using Eve Technologies’ Human Cytokine 71-Plex Discovery Assay® (HD 71) which consists of two separate 48-plex and 23-plex kits (MilliporeSigma). The assay was run according to the manufacturer’s protocol. Individual analyte sensitivity values are available in the MILLIPLEX® MAP protocol.

For flow cytometry, PBMCs were isolated by a Ficoll density gradient on the day of collection before being cryopreserved in 10% dimethyl sulfoxide (DMSO) and 90% fetal bovine serum (FBS). Cells were thawed and distributed at 100,000 cells/well into a 96-well round bottom plate for downstream flow cytometric staining. Cell viability was assessed using LIVE/DEAD Fixable Blue Stain (Life Technologies) alongside staining with an antibody panel (Supplementary Table 1). Samples were stained and washed in PBS with 2% FBS prior to intracellular staining using the eBioscience™ Foxp3/Transcription Factor Staining Buffer Set (Invitrogen) following manufacturer’s instructions. Following, cells were washed twice before being resuspended in PBS with 2% FBS and analyzed using a BD LSRII flow cytometer.

### Statistical analysis

Statistical analyses were carried out using R and Stata. Significance was determined for *p* values less than 0.05.

#### Interim analysis, response analysis, and survival analysis

A two-stage Simon’s optimal design was used. In the first stage, ORR at 12 weeks was assessed compared to a ORR ≤ 60% using a one-sided test with 5% level of significance and a power of 80%. Failure to reach this threshold would have implied an ineffective drug and would have led to early termination. The ORR at the time of the interim analysis was 73% and the study was continued. The study was then powered for a second stage with an ORR > 80%, to represent an effective drug, worthy of pursuing in further trials. However, an additional, not-pre-specified interim analysis was performed at 27 subjects because of the limitations in research capabilities associated with the COVID-19 pandemic. At this point the 12 week ORR was 62.9%, and it was deemed statistically unlikely that the pre-specified end-point of an ORR > 80% for the second stage would be met. Thus, the trial was closed early.

#### Cytokine analysis

In an exploratory analysis, cytokine/chemokine/growth factor concentrations were compared from baseline to on treatment on the day of, but prior to, the cycle 2 infusion (termed pre-cycle 2) and similarly on the day of, but prior to cycle 3 infusion (pre-cycle 3) within each individual using a paired Wilcoxon signed-rank test. The same cytokine/chemokine/growth factor concentrations were then compared between responders (partial or complete response) and non-responders (progressive disease or stable disease) at baseline, pre-cycle 2, and pre-cycle 3. Fold change between an individual’s cytokine/chemokine/growth factor concentrations was assessed at baseline and later treatment cycles. Significant differences were determined by Wilcoxon Rank-Sum Test at the specified time-points. Finally, linear regression between baseline cytokine/chemokine/growth factor concentrations and PFS was completed with adjustment for age and sex. Significance was set at a *p* < 0.05. As this represents a hypothesis-generating analysis in this limited sample size, adjustment for multiple comparisons was not completed.

#### Flow cytometry analysis

Data analysis and gating was performed using FlowJo software. As in the cytokine analysis, significant differences were determined by Wilcoxon Rank-Sum Test using the specified time-points (baseline and pre-cycle 2 or the fold change between these two time-points). For this hypothesis generating analysis, *p* < 0.05 was deemed significant.

## Results

From April 2018 to January 2020, 27 patients with advanced or metastatic melanoma were enrolled to receive combination ipilimumab, pembrolizumab, and high-dose aspirin, with 26 patients completing at least one dose of the triplet combination (Table 1). Prior to enrollment, five patients (18.5%) had received systemic therapy in the adjuvant setting, including two patients treated with adjuvant ICI therapy over six months prior to study enrollment.

**Table 1:** Patient characteristics of 27 patients enrolled to receive pembrolizumab, ipilimumab, and high-dose aspirin for treatment of advanced melanoma.

Characteristic	Enrolled Patients receiving ≥ 1 dose (*n* = 27)
Age, years	
Median	63
Range	31–88
Sex, No. (%)	
Male	16 (59)
Female	11 (41)
*BRAF* Status, No. (%)	
Mutant (V600E)	12 (48)
Wildtype	13 (52)
Unknown	2 (7)
AJCC Stage, No. (%)	
III (unresectable)	5 (19)
IV	22 (81)
M1a	8 (36)
M1b	8 (36)
M1c	6 (27)
M1d	0
Melanoma Subtypes, No. (%)	
Cutaneous	21 (78)
Mucosal	1 (4)
Acral	1 (4)
Unknown Primary	4 (15)
ECOG status, No. (%)	
0	27 (100)
1	0 (0)
Baseline LDH, No. (%)	
≤ULN	20 (74)
≥ULN	5 (19)
≥2 x ULN	2 (7)
CNS Metastasis, No. (%)	
Yes	0 (0)
No	27 (100)
Liver Metastasis, No. (%)	
Yes	5 (19)
No	22 (81)
Prior systemic therapy, No. (%)	
No prior systemic therapy	22 (81)
*BRAF* inhibitor (vemurafenib/cobimetinib)	1 (4)
Adjuvant Interferon	1 (4)
Adjuvant anti-PD1 or CTLA4	2 (7)
Other (Imatinib)	1 (4)

By the data cut-off date of September 22, 2022 the overall median follow-up was 32 months [interquartile range (IQR) 14–45 months] but for those still alive at that date, median follow-up time was 43 months (IQR 42–47 months). Ten patients (37%) discontinued the study due to clinical or radiographic evidence of progressive disease (Supplementary Fig. 1). Other reasons for study discontinuation were adverse events (two patients, 7%), elective withdrawal of consent (two patients, 7%), and death not related to study treatment (two patients, 7%) (Supplementary Fig. 1). Fifteen patients (56%) completed the induction period of four cycles of ipilimumab and pembrolizumab alongside daily high-dose aspirin therapy. Five patients (18.5%) discontinued all treatment prior to completing four cycles of triplet therapy due to abdominal pain, gastrointestinal upset, colitis or disease progression. Six patients (22%) discontinued only aspirin but continued on ICI treatment. One patient never started aspirin, but received ICI treatment, due to history of GI bleed and patient concern about repeat bleeding.

### Efficacy

**Fig. 1:**
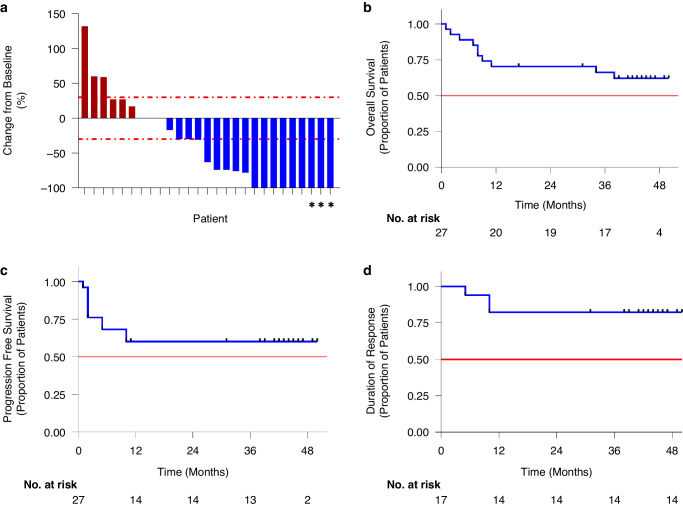
Treatment response and survival outcomes to combination ipilimumab, pembrolizumab and high-dose aspirin. **a** Waterfall plot showing change in tumor volume from baseline at time of best response measured through imaging as defined by RECIST 1.1 criteria. For patients denoted with an *, imaging showed marked partial response, and biopsy of remaining sites revealed only necrotic tissue and no viable malignant cells; therefore, they were considered to be complete responders. Two patients were unable to be imaged prior to death related to disease and were assumed to have progressive disease, they were given a change from baseline value of 0%. Survival curves of OS (**b**), PFS (**c**), duration of response (**d**) as demonstrated by the Kaplan–Meier method. The horizontal line at probability of 0.5 in the figures represents the median survival, which was not reached for either OS, PFS or duration of response. Data shown is for 27 patients enrolled in the study.

Seventeen of 27 patients achieved an objective response (62.9%), with nine complete responses (33.3%) and eight partial responses (29.6%; Supplementary Table 2 and Fig. 1a). Two patients were unable to be assessed for radiographical response due to death assumed secondary to disease progression prior to re-staging and were therefore considered to have progressive disease. PFS at 3 years was 57.9% (95% confidence interval {CI} 0.417–0.803) and OS at 3 years was 66.2% (95% CI 0.505–0.869; Fig. 1b, c). For those patients that responded, the median duration of response was not reached (Fig. 1d). At the time of data cut-off, the median PFS and OS were also not reached.

**Fig. 2:**
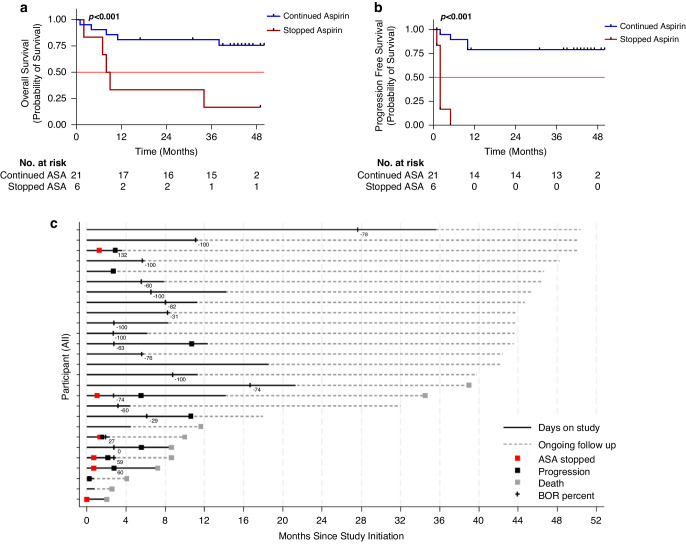
Continuation of high-dose aspirin alongside combination immune checkpoint inhibitors improves survival. Survival curves of melanoma patients who stopped aspirin alone prior to completion of four cycles compared to those that continued aspirin or were taken off study due to progression or death. All patients who stopped aspirin alone stopped for TRAE. OS (**a**), and PFS (**b**) are estimated by the Kaplan-Meier method. **c** Swimmer’s plot showing the time of aspirin cessation for those that stopped aspirin alone (red box), time of progression (black box), death (gray box), time on study (solid black line). It also depicts time of best overall response with percent change in tumor burden shown as a number. Data shown is for 27 patients enrolled in the study. Abbreviations: BOR Best overall response, ASA aspirin, DODC Date of death or censor.

Given the high rate of aspirin discontinuation, we performed an exploratory per-protocol analysis of OS and PFS for those that stopped aspirin due to aspirin attributable adverse events (abdominal pain, gastrointestinal upset, colitis) prior to completing the first four cycles of ICI treatment (Fig. 2a–c). To perform this analysis, patients were divided into those that stopped aspirin alone (*n* = 6) or those that continued aspirin or stopped all treatment (*n* = 21). We found that the 6 patients who stopped aspirin alone experienced a worse OS (median OS 8.5 months, *p* < 0.001; Fig. 2a) and PFS (median PFS 2.76 months, *p* < 0.001 vs median PFS not reached; Fig. 2b) compared to those that either continued aspirin or discontinued all treatment (median OS not reached), despite having similar demographic profiles (Supplementary Table 3). Confirming these results, a time-varying cox regression model, which accounts for survival bias, showed that patients on aspirin had a 0.24 fold hazard (0.07–0.87, *p* = 0.03) of progression relative to those that discontinued aspirin alone.

### Safety

The most common treatment-related adverse events (TRAEs) of any grade were fatigue (17 patients, 63%), arthralgias (16 patients, 60%), rash (12 patients, 44%), colitis/diarrhea (12 patients, 44%), and hypophysitis (all of whom had adrenal insufficiency, 8 patients, 30%) (Table 2). In total, 17 patients (63%) developed TRAEs of grade 3 or higher. The two most common TRAEs of grade 3 or higher were hypophysitis (6 patients, 22%) and colitis/diarrhea (12 patients, 44%). Two patients discontinued the trial due to TRAEs of colitis and liver enzyme elevation, respectively.

**Table 2:** Treatment-related adverse events in melanoma patients receiving ipilimumab, pembrolizumab, and high-dose aspirin.

Event	Number of patients (%)
Any TRAE, Any grade	27 (100)
TRAE in > 5 patients, Any Grade	
Fatigue	17 (63)
Arthralgia	16 (60)
Fevers	6 (22)
Hypophysitis/Adrenal Insufficiency	8 (30)
Hypothyroidism	6 (22)
Colitis/Diarrhea	12 (44)
Abdominal Pain	9 (33)
Nausea/Vomiting	9 (33)
Elevated Liver Enzymes	6 (22)
Pancreatitis or Elevated Lipase/Amylase	6 (22)
Anorexia	8 (30)
Rash	12 (44)
Dry skin/mucosa/eyes	7 (26)
Pruritis	7 (26)
Headache	9 (33)
Neuropathy	6 (22)
Cough	5 (19)
TRAE ≥ grade 3	
Any TRAE ≥ grade 3	17 (63)
Hypophysitis/Adrenal Insufficiency	6 (22)
Colitis/Diarrhea	5 (19)
Pancreatitis or Elevated Lipase/Amylase	3 (11)
Hyponatremia	2 (7)
Atrial Fibrillation	1 (4)
Fevers	1 (4)
Headache	1 (4)
Hypokalemia	1 (4)
Sepsis	1 (4)
Abdominal Pain	1 (4)
TRAE resulting in trial discontinuation	
Elevated Liver Enzymes	1 (4)
Colitis/Diarrhea	1 (4)

### Cytokine analysis by response

**Fig. 3:**
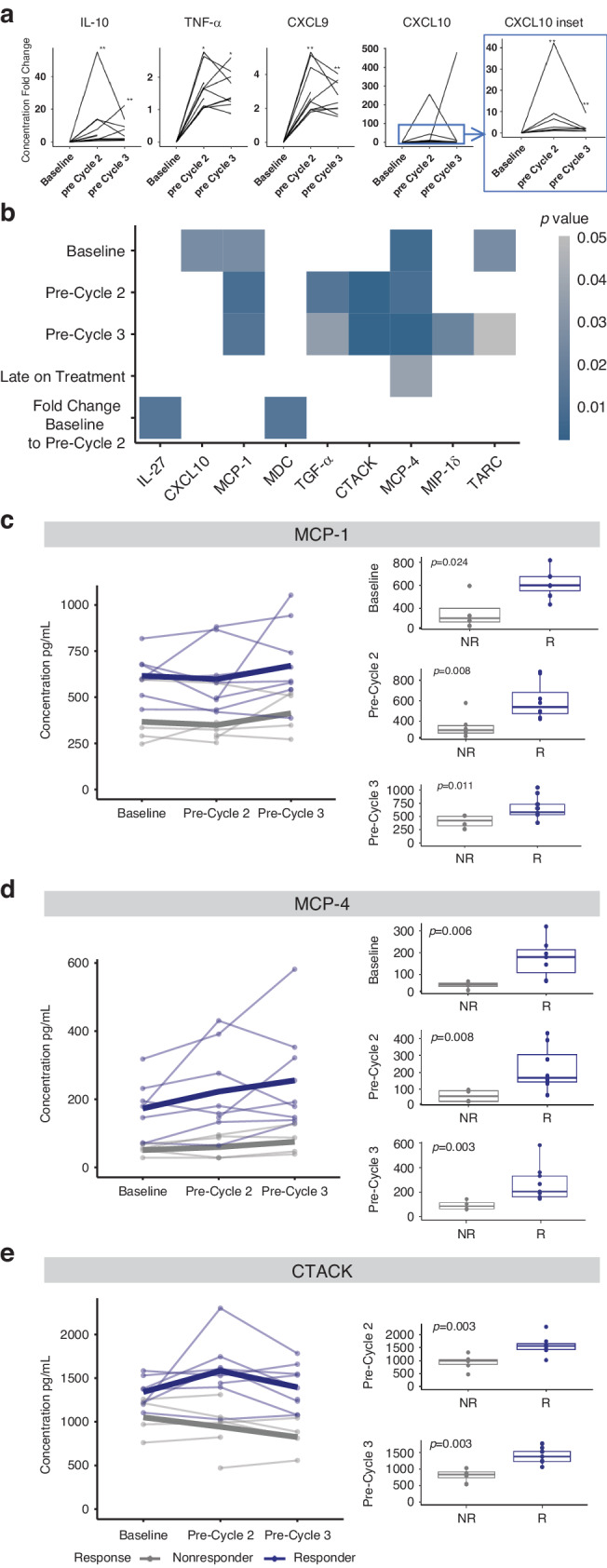
Circulating cytokine profiles associated with therapeutic response. **a** Fold change in cytokine concentrations from baseline to on-treatment timepoints. **b** Heat map of significant cytokine concentrations between responders and non-responders at the specified timepoints was determined using a Wilcoxon Rank-Sum test (cytokines with *p* < 0.05 are shown). Changes over time at baseline, pre-cycle 2, and pre-cycle 3 are shown for responders (dark blue) and non-responders (gray) with the mean expression defined by the thick line for MCP-1 (**c**), MCP-4 (**d**), and CTACK (**e**). Data shown is of (**a**) 14 patients, with (**c**–**e**) showing individual datapoints for individuals who consented to blood collection for research, separated based on response. Changes over time for an individual are shown by lines connecting paired samples across specified time-points. Abbreviations: R responder, NR non-responder, IL interleukin, TNF tumor necrosis factor, CXCL chemokine ligand, MCP monocyte chemoattractant protein, MDC macrophage-derived chemokine, TGF transforming growth factor, CTACK cutaneous T-cell-attracting chemokine, MIP macrophage inflammatory protein, TARC thymus- and activation-regulated chemokine.

With the initiation of triplet therapy, we observed a significant increase in serum concentrations of interleukin (IL)-10, tumor necrosis factor (TNF-α), chemokine ligand 9 (CXCL9), and CXCL10 between baseline and pre-cycle 2 (*p* < 0.001, *p* < 0.050, *p* < 0.001, and *p* < 0.001, respectively) as well as between baseline and pre-cycle 3 (*p* < 0.005, *p* < 0.050, *p* < 0.001, *p*  < 0.001, respectively) (Fig. 3a).

We then compared differences in cytokine concentrations between responders and non-responders at baseline, on-treatment time-points, as well as the fold change between matched baseline and on-treatment timepoints, and identified significant differential concentrations (Fig. 3b–e, Supplementary Fig. 2, Supplementary Table 4). We identified ten cytokines associated with therapeutic response, including MCP-1, MCP-4, CTACK, TGF-α, CXCL10, TARC, MIP-1δ, IL-27, MDC and GRO-α. Preceding treatment initiation, increased concentrations of CXCL10 (*p* = 0.024), MCP-1 (*p* = 0.024), MCP-4 (*p* = 0.006) and TARC (*p* = 0.024) were found in patients that went on to respond to treatment (Fig. 3b–d, Supplementary Fig. 2A) and higher levels of TARC, CXCL9 and GRO-α were associated with longer PFS (Supplementary Table 4).

Following treatment exposure, concurrent with the day of the second infusion (pre-cycle 2), we observed increased concentrations of MCP-1 (*p* = 0.008), MCP-4 (*p* = 0.008) and CTACK (*p* = 0.003) in responders while concentrations of TGF-α (*p* = 0.012) were lower in responders compared to non-responders (Fig. 3b–e, Supplementary Fig. 2). Consistent with pre-cycle 2, we observed higher concentrations of MCP-1 (*p* = 0.011), MCP-4 (*p* = 0.003), CTACK (*p* = 0.003) and lower concentrations of TGF-α (*p* = 0.034) at pre-cycle 3 as well as elevated MIP-1δ (*p* = 0.020) in responders compared to non-responders (Fig. 3b–e, Supplementary Fig. 2). When comparing cytokine concentrations between baseline and pre-cycle 2, we observed a significant increase in fold-change for MDC in responders compared to non-responders (*p* = 0.012) whereas the fold change in IL-27 (*p* = 0.012) was higher in non-responders compared to responders (Fig. 3b, Supplementary Fig. 2). Of these significant cytokines, MCP-1 and MCP-4 were significant at all three time points (Fig. 3c, d).

### Flow cytometry by response

**Fig. 4:**
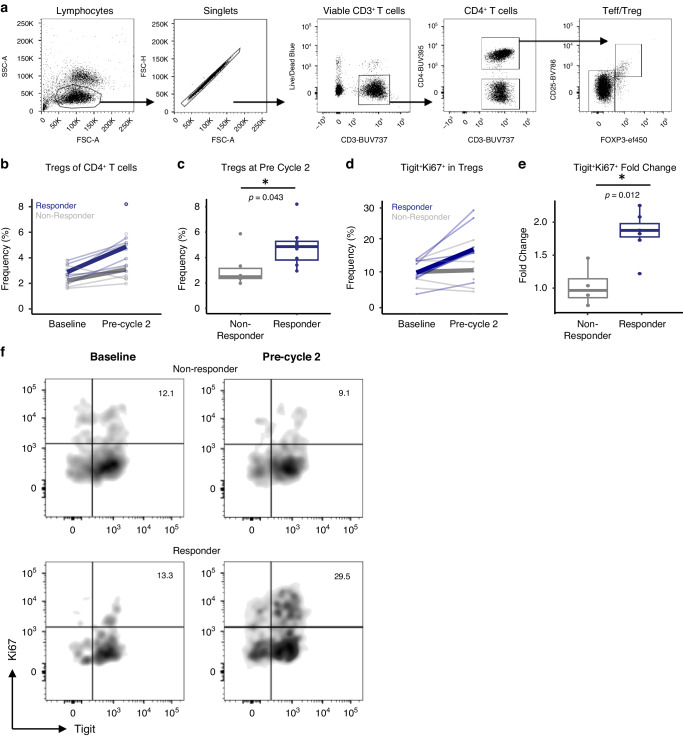
Increased proliferation of T regulatory cells is associated with therapeutic response. **a** Gating strategy used to define T regulatory cells (Tregs) from PBMCs of combination-treated melanoma patients. **b** Frequency of Tregs from CD4^+^ T cells is shown at baseline and pre-cycle 2 for responders (dark blue) and non-responders (gray). Paired samples from an individual across the two time-points are connected by a thin line. Mean frequency is displayed by a thick line. **c** Frequency of Tregs from CD4^+^ T cells at pre-cycle 2 are shown. **d** Frequency of Tregs co-expressing Tigit and Ki67 at baseline and pre-cycle 2 for responders (dark blue) and non-responders (gray). As in B, paired samples from an individual across the two time-points are connected by a thin line. Mean frequency is displayed by a thick line. **e** Fold change in Tigit^+^Ki67^+^ Tregs between baseline and pre-cycle 2 are shown for responders and non-responders. **f** Representative flow cytometric analysis of Tigit^+^Ki67^+^ Tregs relating to response at the specific time-points as shown. Data shown is of individual datapoints for responders and non-responders. Changes over time for an individual are shown by lines connecting paired samples across time-points. *p < 0.05 determined by Wilcoxon Rank-Sum Test at the specified time-points.

We performed flow cytometric analysis to determine if the peripheral immune cell composition displayed changes in proportion or functional status of different immune cell populations to determine correlations with response (Fig. 4a). Responders displayed increased proportions of T regulatory cells (Tregs) at pre-cycle 2, but not at baseline, compared to non-responders (*p* = 0.043; Fig. 4b, c). Consistent with this, responders displayed an increase in the fold change of Tregs expressing the proliferative marker Ki67 as well as the activation/exhaustion marker TIGIT between baseline and pre-cycle 2 compared to non-responders (*p* = 0.012; Fig. 4d–f).

## Discussion

Despite the clinical success of ICI treatment, the presence of multiple immunosuppressive mechanisms can limit anti-tumor immunity [16–21]. Zelenay et al. showed that in a preclinical melanoma model, prostanoids induced a suppressive myeloid response that dampened interferon-γ-induced immune activation and that conversely COX inhibition with aspirin could reverse this suppressive environment [11]. We tested this concept clinically in patients with advanced/metastatic melanoma through treatment with high-dose aspirin combined with pembrolizumab and ipilimumab. To our knowledge, this is the first clinical trial to test this hypothesis prospectively in cancer patients.

In this trial the ORR was 62.9% and the median OS and PFS was not reached despite extended follow-up (>48 months). While these results are generally positive, they are not appreciably different from trials with combined ICI treatment in the literature [6, 7]. In particular, KEYNOTE-029, a phase II trial of the same ICI regimen without high-dose aspirin, showed ORRs of 55–61% [22, 23]. These results indicate that aspirin may not improve survival when added to combination ICI treatment. We also noted a higher rate of TRAEs than has been previously described [5–9]. In the KEYNOTE-029 trials, 22–41% of melanoma patients developed grade 3 or higher TRAEs, with 25% experiencing diarrhea and <4% devleoping hypophysitis/adrenal insufficiency [22, 23]. We observed grade 3 or higher TRAE in 63%, including colitis/diarrhea in 19%, and hypophysitis in 22% of patients. Toxicities were also a major factor in limiting patient’s ability to stay on aspirin, despite the fact that patients were receiving concurrent PPIs. One possible explanation for the higher-than-expected rates of toxicity could be direct effects of aspirin. Aspirin has been shown to affect the function of the hypothalamic-pituitary-adrenal axis and has also been linked to the development or worsening of inflammatory bowel disease [24–30].

We noted a high rate of aspirin discontinuation due to toxicity (6/27 patients were unable to tolerate aspirin within the induction period, leading to cessation of high dose aspirin alone). Because of this high rate of discontinuation, we performed a per-protocol analysis of PFS and OS for those that stopped aspirin alone in the first 4 cycles due to aspirin attributable toxicity compared to those who continued aspirin or discontinued all treatment due to progression or toxicity. These analyses did show that patients who discontinued aspirin alone had shorter PFS and OS compared to those who did not, including a time-varying cox analysis that would account for survival bias, indicating that high-dose aspirin (or potentially a different COX inhibitor with greater specificity) may improve outcome if it could be tolerated. It should be noted that patients in this per-protocol analysis who stopped aspirin, stopped it due to aspirin attributable toxicity as determined by the investigator although it could be difficult in some circumstances differentiating between aspirin alone versus ICI-induced toxicity. However, the high rate of toxicity in this trial compared to traditional ICI without COX inhibition does suggest at least some additive toxicity from aspirin. Future efforts to combine COX inhibition with ICI must therefore focus on either assessing the efficacy of a lower, more tolerable dose or improving the tolerability of a high dose, possibly with medical prophylaxis of gastrointestinal symptoms.

There are some notable limitations to this trial. As a phase II trial, this study was not powered to detect small differences in efficacy, which may have occurred. It is also possible that the preclinical aspirin data studies may not model the complexity of human melanoma adequately [11]. For example, the preclinical models combine COX inhibition with PD1 inhibitors, while in our trial, we added ipilimumab. While this addition was intended to offer patients the aggressive treatment for advanced/metastatic melanoma we may have inadvertently altered or negated the effects of COX inhibition. It is also possible that establishing a predictive threshold for COX2 expression in the TME may allow for selection of patients more likely to benefit. Finally, it is plausible that aspirin was not the ideal COX inhibitor and that a more selective COX2 inhibitor such as celecoxib might have proven more efficacious; however, in preclinical models, the use of aspirin combined with PD-1 inhibition showed increased tumor regression compared to celecoxib and it is unclear that the toxicity profile would be adequately ameliorated [11].

Cytokines may highlight differences in immune activity in ICI-treated cancer patients as they play a critical role in anti-tumor immunity and activation of specific immune subsets within the TME [31]. Within our cohort, our exploratory analysis showed increases in IL-10, CXCL9, and CXCL10 following exposure to treatment across all patients consistent with prior reports of on-treatment changes in these cytokines [32]. We identified 10 cytokines associated with response and/or PFS: CXCL10, TARC, TGF-α, CTACK, MIP-1δ, IL-27, MDC, MCP-1, MCP-4, and GRO-α. Prior analysis of cytokines in patients treated with ICIs (either anti-PD1 alone or combined with anti-CTLA4) has shown an association with baseline levels of MCP-1, MCP-4, and TARC and overall survival or response on imaging by RECIST [33].

These cytokine results offer insight into potential mechanisms for effect of high-dose aspirin alongside ICI treatment. For example, COX enzymes promote differentiation of tumor-associated macrophages towards an immunosuppressive phenotype rather than a pro-inflammatory phenotype, which may limit an effective T helper 1 immune response [11, 34]. We noted that MCP-1 and MCP-4, both cytokines that play an important role in macrophage recruitment, were significantly higher in responders across multiple time-points both preceding and after initiation of treatment [35]. Prior studies of murine melanoma cells producing MCP have demonstrated an increase in tumor-associated macrophages as well as a decreased tumor growth [36]. Similarly, MCP-1-deficient mice displayed reduced melanoma tumor growth, which was due to decreased lymphocyte infiltration that was reversible upon injection of MCP-1 [37]. While the data on MCP-1 specifically show model-dependent effects, higher levels of MCP-1 secretion in mice has been associated with increased macrophage/monocyte infiltration of melanoma tumors, leading to increased tumor necrosis [38–40]. Given that this is a single-arm trial, we are unable to determine whether these changes may be specific to COX inhibition and the previously observed relationship for skewing macrophages towards a pro-inflammatory phenotype and therefore a more effective anti-tumor immune response. Nonetheless, this highlights that understanding the connection between strategies to augment this cytokine profile and their relationship to response may yield beneficial outcomes for melanoma patients.

Prior analysis has also has demonstrated a link between COX enzymes and suppression of cytotoxic T cell response, thus leading to tumor immune invasion [41]. We found that the cytokine CTACK was significantly associated with response. Pre-clinical models suggest that CTACK is upregulated by inflammatory cytokines, including IL-1 and TNF-α, and involved in the recruitment of both CD8^+^ T cells and effector memory T cells, thus possibly potentiating the cytotoxic response to melanoma tumor cells [42, 43]. Interestingly, CTACK and its receptor CCR10 have been shown to be upregulated in melanoma tumors and linked to inflammatory skin disorders such as psoriasis, atopy, and allergic-contact dermatitis, indicating that it may be a critical mediator of T cell-mediated skin inflammatory disorders [44]. Again, this single-arm trial is unable to distinguish between the sole effect of COX inhibition to promote CTACK compared to ICI alone, but may point to an important mechanism of cytotoxic T cell recruitment which may further augment the anti-tumor immune response.

Within our cohort, we performed an exploratory analysis in which we observed increased proliferation of activated Tregs correlating with response. This trend appears to be consistent with other ICI-treated cancers [45]. Interestingly, prior evaluation of resected non-small cell lung cancer tissue has shown a link between COX2 expression and intra-tumoral Tregs, indicative that it will be important to determing the influence of aspirin towards Treg function in future studies [46]. While a correlation between response and higher peripheral Treg frequency may appear counterintuitive, it could be in response to heightened immune activation, thereby acting as an indirect marker of better survival outcomes, much the way immune-related adverse events have been associated with ICI response.

## Conclusion

In summary, our phase II clinical trial provided insufficient evidence that the addition of high-dose aspirin to combination ICI treatment improves response in advanced/metastatic melanoma. However, we do note a high rate of toxicity with the addition of high-dose aspirin, limiting our assessment of treatment efficacy. Per-protocol analysis demonstrated that patients that did not discontinue aspirin through the induction period due to aspirin-related toxicity did experience improved survival benefit. These results indicate that the use of high-dose aspirin with combination ICI will require consideration of aspirin dose and/or aggressive toxicity management. Cytokines significantly associated with therapeutic response suggest alterations to the immune response that may enhance the function of both macrophages and CD8 + T cells. Changes in immune cell composition suggest increased Tregs, which could be an indicator of improved immune activation. Finally, further investigation is needed to fully demonstrate the clinical utility and tolerability of COX inhibition when combined with ICIs.

## Supplementary information

Supplementary File

## Data Availability

The datasets generated during and/or analysed during the current study are available from the corresponding author on reasonable request.
