# Supplementary material for: Phase II trial of pembrolizumab, ipilimumab, and aspirin in melanoma: clinical outcomes and translational predictors of response
Source: BJC Rep. 2024 Jun 24;2:46. doi: 10.1038/s44276-024-00057-7 (PMC11524064; doi:10.1038/s44276-024-00057-7)
Supplement: Supplementary file 1 — Supplementary File [file 44276_2024_57_MOESM1_ESM.docx]

SUPPLEMENTARY TABLES

| Supplementary Table 1. Antibody panel for flow cytometry | | |
| --- | --- | --- |
| REAGENT | SOURCE | IDENTIFIER |
| Cell surface staining: |  |  |
| LIVE DEAD Fixable Blue | Thermo Fisher Scientific | L23105 |
| Anti-human CD3, BUV737 (clone UCHT1) | BD Biosciences | 564308 |
| Anti-human CD4, BUV395 (clone SK3) | BD Biosciences | 563550 |
| Anti-human CD8a, PerCP-Cy5.5 (clone RPA-8) | BD Biosciences | 560662 |
| Anti-human CD25, Brilliant Violet 786 (clone M-A251) | BD Biosciences | 557138 |
| Anti-human CD39, PE-Cy7 (clone A1) | BioLegend | 328212 |
| Anti-human CD45RO, Brilliant Violet 605 (clone UCHL1) | BD Biosciences | 304238 |
| Anti-human KLRG1, PerCP-Cy5.5 (clone 2F1/KLRG1) | BioLegend | 138417 |
| Anti-human TIGIT, APC (clone A15153G) | BioLegend | 372706 |
| Intracellular staining: |  |  |
| Anti-human FOXP3, efluor 450 (clone 236A/E7) | Thermo Fisher Scientific | 48-4777-42 |
| Anti-human Granzyme B, FITC (clone GB11) | BD Biosciences | 561998 |
| Anti-human Ki67, PE (clone Ki-67) | BioLegend | 350504 |

| **Supplementary table 2.** **Objective Response at Time of Best Response** | |
| --- | --- |
| **Response** | **No. (%)** |
| Complete Response | 8 (29.6) |
| Partial Response | 7 (25.9) |
| Stable Disease | 5 (18.5) |
| Progressive Disease | 7 (25.9) |

| **Supplementary Table 3.** Representativeness of Study Participants | |
| --- | --- |
| Cancer type(s)/subtype(s)/stage(s)/condition | Melanoma |
| Considerations related to: | |
| Sex | Melanoma is known to have higher incidence in men compared to women with the American Cancer Society estimating that of the new cases in 2023, about 58,120 will be men and 39,490 women. |
| Age | The average age at the time of melanoma diagnosis is 65. |
| Race/ethnicity | Melanoma is most common in non-hispanic whites, making up >90% of all melanoma cases. |
| Geography | Estimated new cases of melanoma in California for 2023 are 10,950 with 690 estimated deaths. |
| Other considerations | While melanoma is most common in non-hispanic whites, there is some data to suggest that the proportion of cases diagnosed at Stage III/IV was twice as high for Black and Native American patients compared to White patients. |
| Overall representativeness of this study | The age distribution of our study is similar to the average age distribution of melanoma in the literature, median age of . |

SUPPLEMENTARY FIGURES

**Supplementary Figure 1. CONSORT flow diagram of the single-arm phase II trial.**


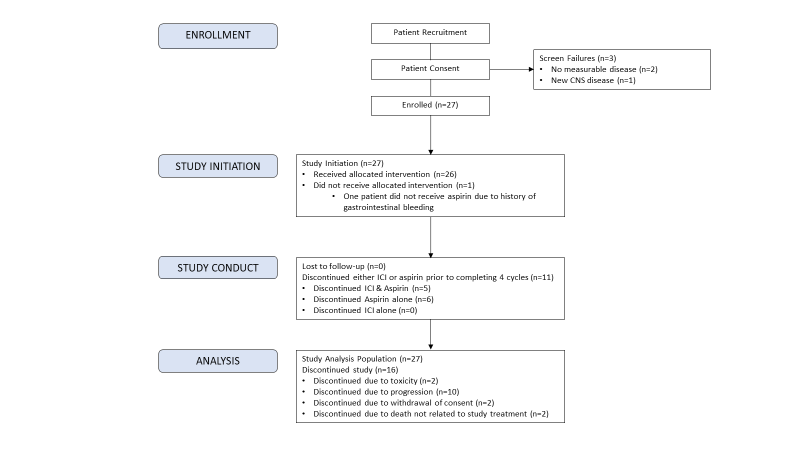


**Supplementary Figure 2**


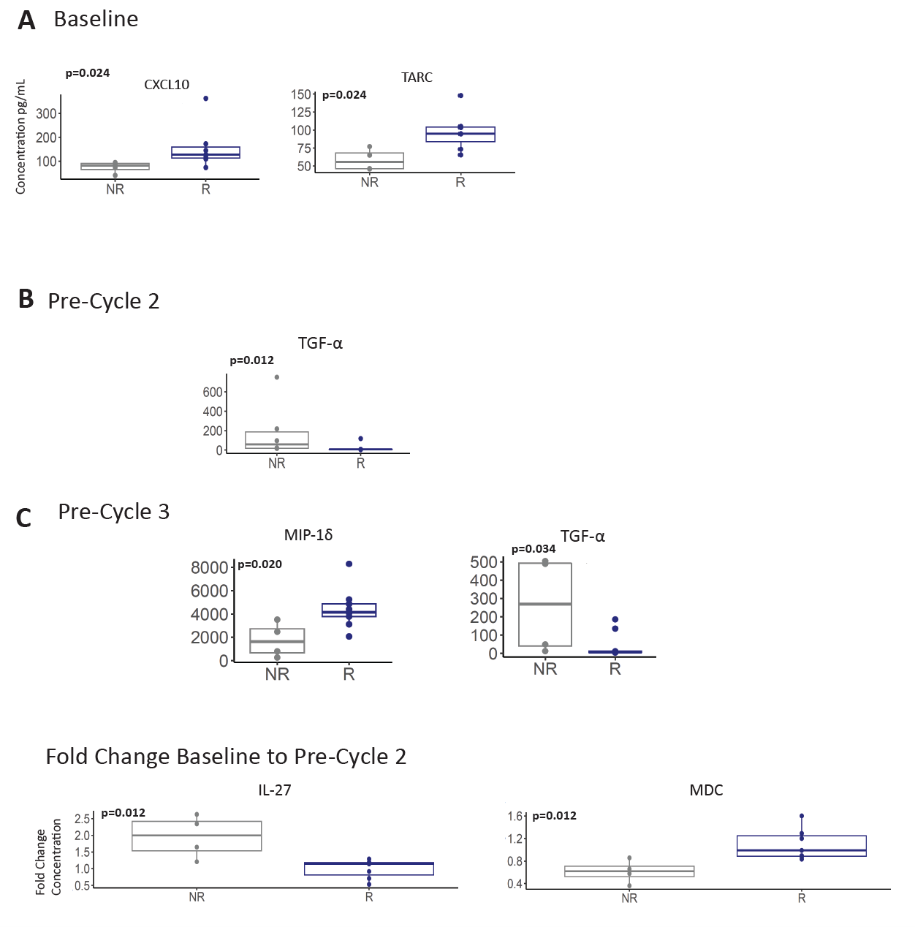


**Supplementary Figure 2. Circulating cytokine profiles associated with response. (A)** Cytokines were compared at baseline between responders and non-responders with responders having higher concentrations of CXCL10 and TARC. **(B)** Cytokine analysis at pre-cycle 2 showed lower levels of TGF-α in responders. **(C)** At pre-cycle 3, responders had higher levels of MIP-1δ and lower levels of TGF-α. **(D)** Fold change was calculated for paired samples of an individual between baseline and pre-cycle 2with significant changes shown.
